# Evidence of a High Density Population of Harvested Leopards in a Montane Environment

**DOI:** 10.1371/journal.pone.0082832

**Published:** 2013-12-09

**Authors:** Julia N. Chase Grey, Vivien T. Kent, Russell A. Hill

**Affiliations:** 1 Department of Anthropology, Durham University, Durham, United Kingdom; 2 Primate and Predator Project, Lajuma Research Centre, Louis Trichardt (Makhado) , South Africa; 3 Durham Wildlife Trust, Houghton-le-Spring, United Kingdom; Bangor University, United Kingdom

## Abstract

Populations of large carnivores can persist in mountainous environments following extensive land use change and the conversion of suitable habitat for agriculture and human habitation in lower lying areas of their range. The significance of these populations is poorly understood, however, and little attention has focussed on why certain mountainous areas can hold high densities of large carnivores and what the conservation implications of such populations might be. Here we use the leopard (*Panthera pardus*) population in the western Soutpansberg Mountains, South Africa, as a model system and show that montane habitats can support high numbers of leopards. Spatially explicit capture-recapture (SECR) analysis recorded the highest density of leopards reported outside of state-protected areas in sub-Saharan Africa. This density represents a temporally high local abundance of leopards and we explore the explanations for this alongside some of the potential conservation implications.

## Introduction

Carnivore density and distribution is limited by a range of ecological and anthropogenic factors. One of the main constraints of carnivore density is prey availability and abundance, with carnivore densities being positively correlated with the density of their prey [1,2]. Large carnivores also require sufficient suitable habitat to provide cover for hunting and females require denning sites for rearing their young [3,4]. Anthropogenic factors limiting carnivore density include habitat loss [5], decline of prey densities due to hunting by humans [6], human persecution [7] and unsustainable harvest levels [8].

Widespread conversion of habitat into land for cultivation and human habitation has resulted in extensive habitat loss for many large carnivores. As a consequence, populations of felids, such as leopards (*Panthera pardus*) and pumas (*Puma concolor*), occur in mountainous areas that are less accessible to humans, where they persist after extirpation from lower lying altitudes of their ranges [9-15]. The conservation significance of these populations is poorly understood.

The leopard is one of the most widely distributed of the Felidae and inhabits a broad range of different habitats [9,16]. Their large geographic range is partially explained by their highly adaptable feeding behaviour which allows them to live wherever there is a sufficient prey base and hunting cover [17,18]. Nevertheless, global population numbers have declined over the last 100 years [19] and leopards have disappeared from 36.7% of their historical range in Africa [5]. The species was recently reclassified by the IUCN (International Union for Conservation of Nature) as “Near Threatened” and some of the leopard’s most dramatic range loss has occurred in South Africa [9,20]. Decline of leopard habitat and populations can be due to habitat loss and fragmentation [5], poorly managed harvest quotas [8,21] and persecution [16,20]. Furthermore, there is a lack of widespread scientific input in quota setting for legal trophy hunting in many countries and few data exist on leopard numbers or metapopulation dynamics in many areas in which they are hunted [22]. Here we address the need for more data on leopard populations subject to both legal and illegal hunting and persecution through an examination of the population status of the leopard in the western Soutpansberg Mountains, South Africa. In identifying the existence of a temporally high population of leopards in the mountains, we discuss the factors contributing to this high density and the implications for conservation management and metapopulation dynamics.

## Materials and Methods

### Ethics Statement

All fieldwork was approved by the Life Sciences Ethical Review Process Committee at Durham University, UK, and the Department of Anthropology Ethics Committee, with the ethics guidelines of the Association of Social Anthropologists of the UK and Commonwealth adhered to when interviewing landowners. All work was conducted with approved permits from the Limpopo Department of Economic Development, Environment & Tourism, South Africa.

### Study area

**Figure 1:**
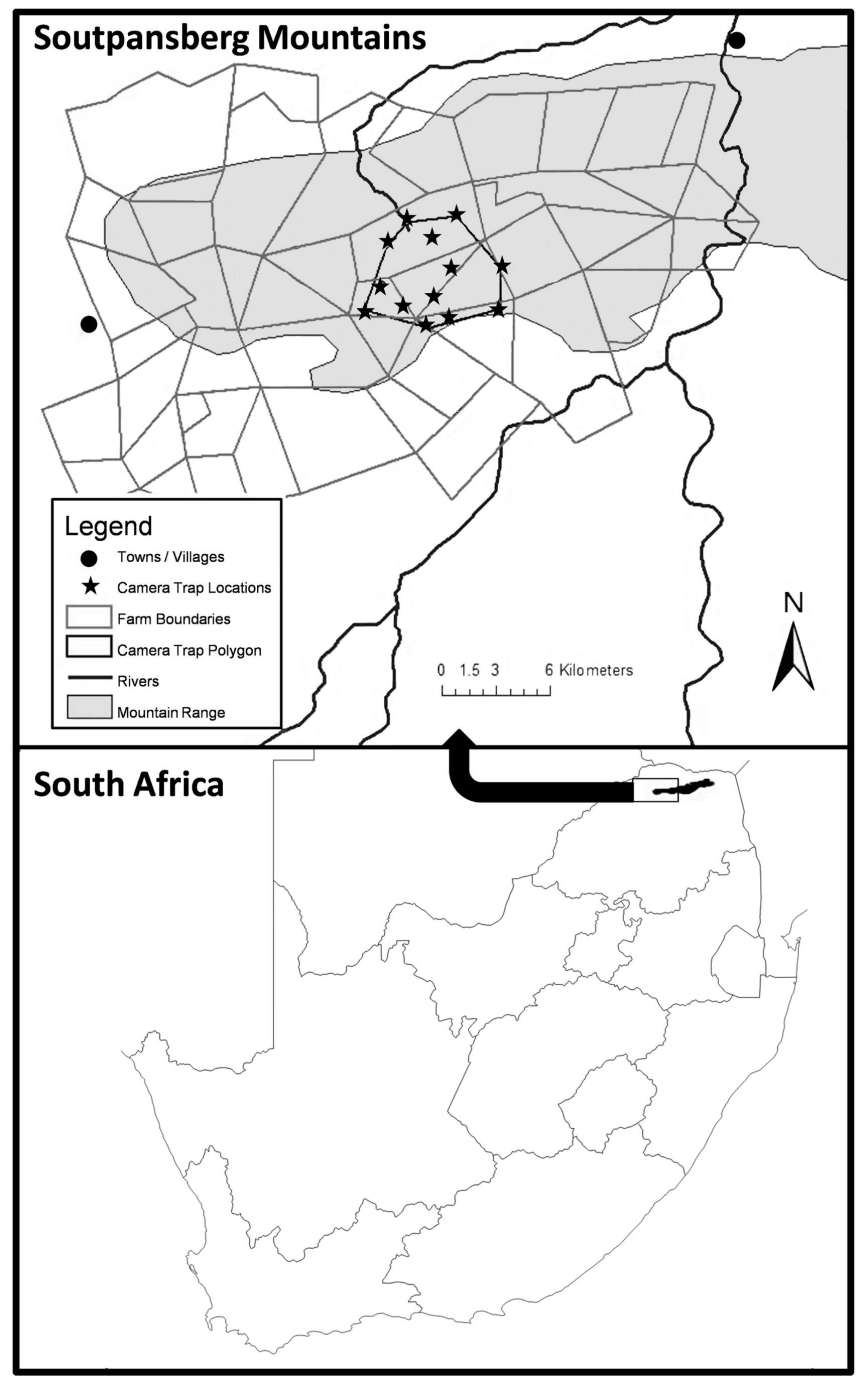
Camera trap study area within the western Soutpansberg Mountains. Lower map shows the location of the mountain range in Limpopo Province, South Africa.

The study was conducted in the western part of the afro-montane forests of the Soutpansberg Mountains, Limpopo Province, South Africa (Figure 1). The mountains cover approximately 600km^2^ and range in height from 250m above sea level to the highest peak Letjume (1748m) at the western extremity [23]. Temperatures vary in the wet season (December-February) from 16-40°C and in the dry season (May-August) between 12 and 22°C [24]. The western Soutpansberg is part of the Vhembe Biosphere Reserve, recognised as a hotspot of South African biodiversity and endemism [25].

Land use in the western Soutpansberg consists of a patchwork of private cattle and game farms, ecotourism properties, conservancies and communal farm land. Uncontrolled hunting during the 19^th^ century and the destruction of habitat from cattle farming led to the extinction of mammals such as the African elephant (*Loxodonta africana*) and the black rhinoceros (*Dicero bicornis*) [26,27]. The only large carnivore species that remain resident in the western part of the mountain range are leopards, brown hyaena (*Parahyaena brunnea*) and spotted hyaena (*Crocuta crocuta*) [28]. 

### Sampling design and field methods

Leopard population density was determined through the application of a Bayesian spatially explicit capture-recapture (SECR) model to data acquired through camera trapping, a population sampling technique that allows researchers to estimate population densities of species that can be individually recognised from their natural markings [29,30]. Since the development of camera trapping, the methodology has been used extensively to obtain density estimates for a wide range of carnivores [31-38].

A camera trapping survey was carried out from March to May 2008. Following a two month scat and track survey, 13 paired camera trap stations were set up over an area of 31km^2^ along roads and trails known to be frequented by leopards [39]. Camera stations were set up at a minimum distance of 1.7 km and a maximum distance of 3.5 km in order to ensure that all individuals in the study area had a probability of capture and that there were no gaps large enough to contain the home range of an individual leopard [39]. An area of 9 km^2^ was used as the smallest recorded home range size for an adult female leopard in a forested habitat [40] and so, to ensure even camera coverage, at least 2 camera trap pairs were positioned within an area of this size [31]. 

Each camera trap station consisted of a pair of Cuddeback® Expert Digital Scouting Cameras (Non Typical Inc, USA) placed facing each other on either side of a road or trail. The cameras in each pair were placed a maximum of 6 metres apart to ensure that photographs of entire leopards were obtained and at a height of 40cm corresponding approximately to the shoulder height of an adult leopard [31]. Cameras were fixed to trees or set on bamboo stakes and fitted with roofs to protect them from sunlight and adverse weather conditions. The cameras were set to run continuously and were programmed to the smallest delay available for the models (one minute). The mountainous topography of the site made it impractical to move cameras to new positions during the survey and so cameras remained in fixed positions.

In order to meet the assumption of population closure, such that the sampling period is short enough so that no births, deaths, or emigration/immigration occur, the survey lasted for 63 days (9 weeks) [39]. To prevent battery failure, cameras were checked every two weeks with images downloaded from memory cards onto a laptop. Once images were saved, the date, time and location of each photograph was noted. All cameras were functional for the full duration of the study. 

### Spatially explicit capture-recapture camera trapping

Each camera trapping night was used as one trapping occasion. The sex of each photographed leopard was established via the presence of external genitalia and relative body size and individuals were identified via their unique spot patterns. Due to the difficulty of correctly assigning leopards to narrow age categories from photographs [41], individuals were put in two broad age classes, juvenile (less than 2 years old) or adults (above 2 years old) in order to prevent confounding the data [41]. Age classification was conducted by multiple researchers via the examination of three diagnostic characteristics: extent of facial scarring, ear condition and dewlap size for males [41].

A Bayesian SECR model was used to estimate leopard density from photographs of individual leopards and capture locations. SECR provides a more accurate measurement for the effective survey area than solely using capture-recapture analysis as it uses the locations where each animal is detected to fit a spatial model of the detection process [42,43]. SECR obtains estimates of population density unbiased by edge effects, incomplete detection and heterogeneous capture probabilities and eliminates the need for an ad hoc estimation of the sampling area [44-47]. SECR analysis was conducted using SPACECAP version 1.0.6 [48] in R version 2.15.2 [49].

SPACECAP requires three data input files: 1) animal capture details (information on animal identification, trap location and sampling occasion), 2) trap deployment details (spatial location, dates when specific traps were active and sampling occasion) and 3) state-space details. In SPACECAP analyses, the surveyed area containing the camera trap array is combined with an extended area surrounding it, known as the "state-space" of the underlying point process, S, which is represented by a large number of equally spaced points in the form of a very fine mesh. These points are visualized as representing all possible potential activity centres (or home range centres) of all the animals in the population being surveyed [48]. The state space area equals the outer camera trap rectangle surrounded by a large buffer area. ‘S’ was created using ArcGIS 9.3 (ESRI, Redlands, USA) and was made up of UTM coordinates of potential home range centres created as equally spaced points and an associated column indicating habitat suitability at each point. A buffer distance, which is sufficiently large to ensure that no individual animal outside of the buffered region has any probability of being photo-captured by the camera traps in the array during the survey, was added to the rectangle encompassing the trap array. Then, using ArcGIS 9.3, numerous equally spaced points representing home range centres were generated for this extended area [48]. For the state-space file (the buffered area including the camera polygon) the mesh size was set at 1 km. Habitat not thought suitable for leopards (arable areas) was removed from the analysis. The areas removed were large, flat crop circles that provided no hunting cover for leopards. Interviews with local farmers confirmed that leopards were not seen in these areas. The model was run with buffers of 10, 15, 20 and 25 km, both with and without suitable habitat removed in order to examine 1) the effect of the habitat mask on the resulting density estimate [50] and 2) at what point the density estimate stabilised.

The following model definitions were used for the analysis: trap response present, spatial capture–recapture, half-normal detection function and Bernoulli’s encounter model. The ‘trap response present’ option runs the behavioural response option (equivalent to Model "Mb"). This model implements a local or "trap-specific" behavioural response under which the probability of encounter in a trap increases subsequent to initial capture in that trap [48]. The number of Markov-Chain Monte Carlo (MCMC) iterations was set at 100,000 with a burn-in period of 20,000 iterations and a thinning rate of 1. The analysis was run with a data augmentation value of 375 (37.5 times the number of animals identified in the survey) in order to achieve chain convergence for all parameters [51]. Chain convergence was assessed via examination of z score values produced by the Geweke diagnostic statistic in SPACECAP. Z scores greater than 1.6 imply that the MCMC analysis has not been run long enough and chains have not converged. A Bayesian P-value is produced by SPACECAP allowing for assessment of the adequacy of the model, values close to 0 or 1 imply that the model is inadequate [48]. 

### Prey abundance

In order to examine the abundance of prey species in the western Soutpansberg Mountains, an index of species abundance and a relative abundance index were calculated from camera trap photographs taken during the survey. Relative abundance indices from camera trap surveys have been shown to be directly related to independently derived density estimates of these species [32]. 

To calculate prey species abundance, each photograph of an animal was identified to species level and the time and date recorded, with the photo then classified as an independent or dependent event. An independent event was defined as consecutive photos of different species or consecutive photographs of individuals of the same species taken more than 1 hour apart [52]. A RAI was then calculated from the camera trapping data. The RAI equalled the number of days required to obtain a photograph of each species. This index measured effort and was expected to decrease as density increased with a score of 0 indicating that the species was captured on the day the camera trap survey began, required low capture effort and existed at a high density on the study site [53]. Species with higher RAI scores took a greater capture effort to obtain a photograph and therefore signified a lower density. 

## Results

### Leopard density

**Figure 2:**
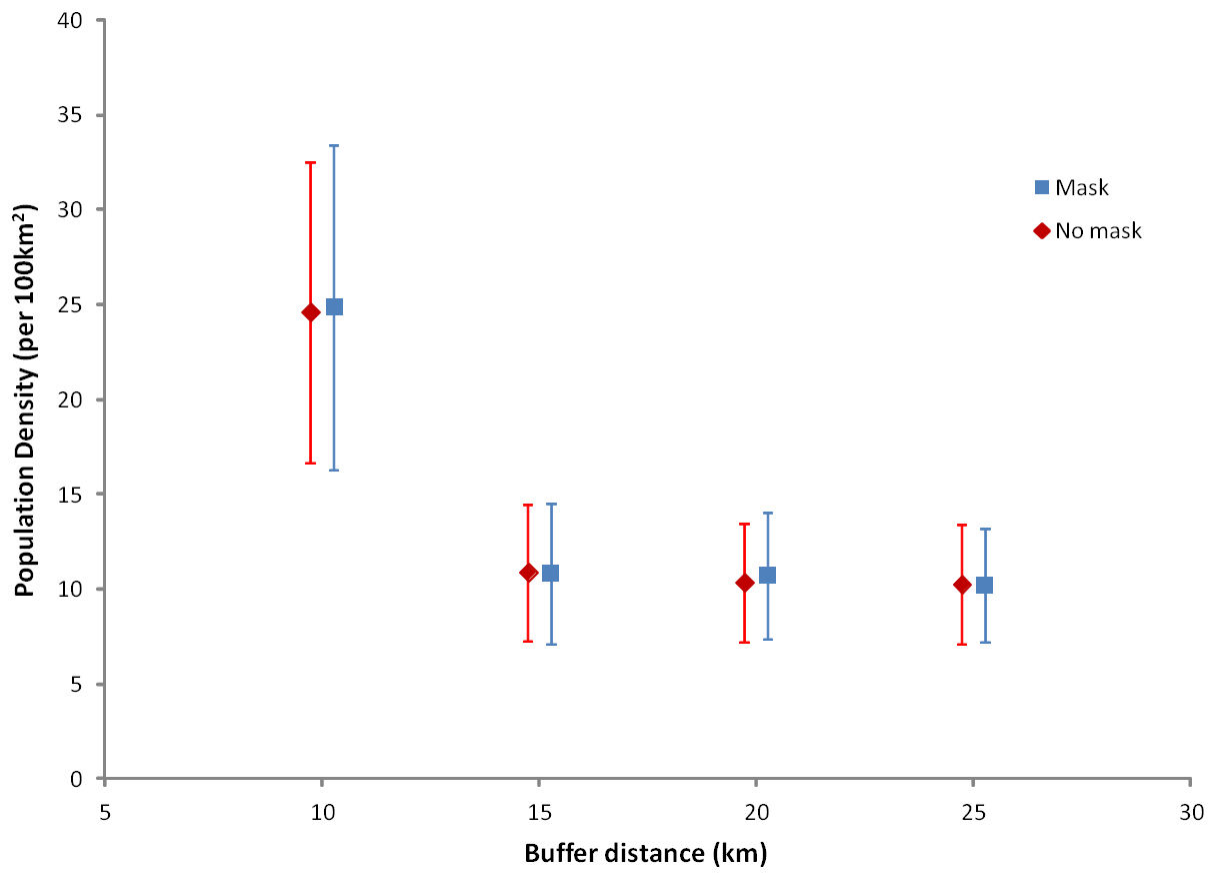
Graph showing the effect of buffer increase and use of a habitat mask on the SPACECAP density estimate (mean and standard deviation).

103 photographs (53 left flanks and 50 right flanks) of 10 individual adult leopards (7 females and 3 males) were obtained over 819 trap nights, alongside 31 photographs (15 left flank and 16 right flank) of 4 juvenile individuals (2 males and 2 females) (Table 1). Capture histories of individually identified leopards were used to calculate capture frequencies. The number of captures and recaptures ranged from 1 to 19. Only adult leopards were used to calculate leopard densities as cubs of solitary felids (age <1 year) have been found to have low capture probabilities and sub-adults may be transient individuals and therefore not part of the resident population [54]. Spatially explicit capture recapture via SPACECAP provided a density estimate of 25 adults per 100km^2^ (SD 8.54) with a buffer of 10km. The density estimates then stabilised between 10.85 (SD 3.68) and 10.22 (SD 3.00) for buffers between 15km and 25km (Figure 2). The removal of unsuitable leopard habitat had little effect on the final result. Since a buffer of 20km is larger than the home range of a female leopard collared in the western Soutpansberg (14km^2^) [55], this buffer was selected to ensure that the buffer area was large enough such that no individual animal outside of the buffered region had any probability of being photo-captured by the camera traps during the survey. With a 20km buffer the mean density estimate was 10.73 adult leopards per 100 km^2^ with a standard deviation of 3.32 (Table 2). Without unsuitable habitat removed from the state space area, the density was 10.41 adult leopards per 100 km^2^ with a standard deviation of 3.13 suggesting that the unsuitable habitat did not significantly influence the density estimates. The density estimate obtained with a 20km buffer and unsuitable habitat removed is thus robust and a Bayesian P value of 0.60 confirmed the adequacy of the model with Z scores from the Geweke diagnostic confirming that chain convergence had been achieved for all parameters (Table 2). The encounter probabilities (posterior means) for individuals pre- and post-initial encounter were p_1_ = 0.0286 and p_2_ = 0.649 indicating a positive trap response. This is unlikely to reflect ‘‘trap-happiness’’, however, but instead indicates non-independence among encounters due to leopards favouring certain trails in moving through their territories [47].

**Table 1:** Individuals and age sex classes identified from camera images in the western Soutpansberg Mountains, South Africa.

**Leopard ID Code**	**Total captures**	**Number of capture locations**
**AF1**	6	3
**AF2**	11	4
**AF3**	4	1
**AF4**	2	1
**AF5**	4	2
**AF6**	1	1
**AF7**	1	1
**AM1**	2	1
**AM2**	10	6
**AM3**	14	3
**JF1**	5	1
**JF2**	4	1
**JM1**	2	2
**JM2**	3	1

AF (adult female), AM (adult male), JF (juvenile female) and JM (juvenile male).

**Table 2:** Posterior summary statistics and z scores from the SECR model fitted to the leopard camera trapping data in the western Soutpansberg Mountains, South Africa.

**Parameter**	**Mean**	**SD**	**95% lower HPD level**	**95% upper HPD level**	**Z scores**
*D*	10.73	3.32	4.68	17.33	
*λ*_0_	0.029	0.0089	0.013	0.047	-0.86
*σ*	1.69	0.26	1.25	2.23	-0.71
*b*1	1.10	0.32	0.46	1.72	-0.22
Ψ	0.54	0.17	0.23	0.87	0.22
*Ns*	206.16	63.81	89.00	332.00	0.22

Only adult leopards were included. *D* is density/100 km^2^; *λ*_0_is the expected encounter rate; *σ*= sqrt(1/b^2^) is the scale parameter of a bivariate normal encounter function and may also be viewed as a range parameter of an animal; *b*1 is the regression coefficient measuring the behavioural response; Ψ is the ratio of the number of animals actually present within the state space to the maximum allowable number; *Ns* is Nsuper and equals the population size for the state space.

### Prey abundance

Table 3 shows the capture frequencies of prey species expressed as number of photos per 100 camera days and the results of the relative abundance index. Bushbuck (*Tragelaphus scriptus*) were the most frequently captured species in camera trap photographs with a capture frequency of 1.56 per 100 traps nights. They were also one of the prey species that took the least measured effort (0 days) to obtain a photograph. Other small to medium sized antelopes known to be preferred leopard prey [3] included impala (*Aepyceros melampus*), red (*Cephalophus natalensis*) and common duiker (*Sylvicapra grimmia*). These species were captured at lower frequencies of 0.31 (impala), 0.17 (red duiker) and 0.12 per 100 trap nights (common duiker). Capture effort for impala and red duiker was relatively low compared to other species (RAI = 3), although the RAI value was higher for common duiker.

**Table 3:** Capture frequencies expressed as number of independent photos per 100 camera days and a relative abundance index calculated from capture effort for 18 species in the western Soutpansberg Mountains, South Africa.

Species	Capture frequency (per 100 camera days)	Relative abundance index (capture effort)
Bushbuck	1.56	0
Porcupine	1.09	3
Baboon	1.08	0
Kudu	0.5	5
Giraffe	0.37	3
Impala	0.31	3
Lesser spotted genet	0.31	9
Warthog	0.31	2
Civet	0.3	5
Helmeted guinea fowl	0.29	0
Aardvark	0.17	2
Red duiker	0.17	3
Cattle	0.14	6
Sable	0.13	6
Common duiker	0.12	7
Donkey	0.12	6
Bush pig	0.1	19
Nyala	0.09	7

## Discussion

The results of this study provide evidence of a high density estimate of leopards in the montane habitat of the western Soutpansberg Mountains (10.7 per 100 km^2^). This density estimate is the highest adult leopard population density recorded outside a state-protected area in Africa (Table 4). Areas of greater leopard density have been reported in the Sabie river area of southern Kruger National Park, South Africa (30.3 leopards per 100km^2^) [56], Phinda Private Game Reserve [7] (11.25 per 100km^2^), the N'wanetsi concession, Kruger National Park (12.7 per 100km^2^) [57] and Ivindo National Park in Gabon (12.1 per 100km^2^) [58], but these all occur in national parks. Our reported leopard density greatly exceeds densities reported in other mountainous areas such as in the Cederberg and Waterberg Mountains, South Africa, where density ranges from 0.62 to 3 per 100km^2^ [12,14]. It is important to note, however, that our study only represents a point density estimate and longer term camera trapping surveys need to be undertaken in order to examine population fluctuations and the effects of factors such as seasonality on leopard population numbers. Nevertheless, our results suggest a significant leopard population within the Soutpansberg Mountains.

**Table 4:** Leopard densities reported for previous studies across sub-Saharan Africa.

**Location**	**Density (per 100km^2^)**	**State-protected**	**Habitat type**	**Reference**
Kruger National Park, South Africa	30.3	Y	Riverine forest	[56]
Ivindo National Park, Gabon	12.1	Y	Forest	[58]
N'wanetsi concession, Kruger National Park	12.7	Y	Savannah woodland	[57]
Phinda Private Game Reserve	11.25	Y	Woodland and grassland	[7]
Western Soutpansberg, Limpopo, South Africa	10.7	N	Montane woodland	This study
Tai National Park, Ivory Coast	8.7	Y	Forest	[76]
Tsavo National Park, Kenya	7.7	Y	Montane bushveld	[77]
Ranches, Laikipia District, Kenya	5.5 - 8.5	N	Savannah and woodland	[78]
Serengeti National Park, Tanzania	4.7	Y	Forest	[79]
Serengeti National Park, Tanzania	3.8	Y	Forest	[80]
North-central farmland, Namibia	3.6	N	Shrub and woodland	[81]
Kruger National Park, South Africa	3.5	Y	Savannah	[56]
Waterburg, South Africa	3.0	N	Montane savannah	[12]
Logging Concession, Gabon	2.7	N	Forest	[58]
Cederberg Wilderness Area, South Africa	2.0	Y	Montane fynbos	[14]
Kaudom Game Reserve, Namibia	1.5	N	Savannah	[82]
Waterberg Plateau Park, Namibia	1.0	Y	Savannah and woodland	[81]
Cederberg Wilderness Area, South Africa	0.62	Y	Montane karoo	[14]
Kalahari Gemsbok National Park South Africa	0.6	Y	Savannah	[83]

There are a number of possible reasons for the high density recorded in the Soutpansberg. Firstly, it may represent a temporally local high abundance of leopards. Leopard population numbers can fluctuate significantly over time and a study in the Karonge Game Reserve, South Africa, found that leopard density changed from 20 per 100km^2^ to 5 per 100km^2^ over a five year period [59]. Population numbers may be affected by short-term changes in the environment and in population dynamics which can cause variance in breeding success [60]. The possibility exists, therefore, that the densities recorded here may not be stable in the long term and further work examining the population dynamics of these leopards is required.

Carnivore abundance is also limited by the biomass of its prey, and variation in leopard density is partly due to differences in biomass and abundance of prey species [1,2]. Leopards preferentially feed upon animals between 10 and 40 kg that live in small herds, occupy dense habitat and afford minimal risk of injury during capture such as bushbuck, common and red duikers [3,55]. Bushbuck, the most frequently taken prey item by relative frequency in leopard diets in the western Soutpansberg [55], was both the most commonly captured species in camera trap photographs and one of the prey species that took the least measured effort to obtain a photograph. This suggests that the survey area holds a high abundance of bushbuck, a preferred leopard prey species, which may account for the high density of leopards on the study site. Although the RAI does provide on information on prey abundance, it was not possible to collect independently derived data on leopard prey species due to the highly mountainous terrain which made conducting line transects impractical [61]. Nevertheless, known preferred prey species such as bushbuck and other small to medium antelope are well represented in our data. Since the RAI provides an indication of the abundance of these species at the survey site it suggests a high prey base of preferred species within the Soutpansberg Mountains. 

A modelling study to estimate the extent of suitable leopard habitat in South Africa found surface ruggedness to be one of the highest contributing variables underlying the most parsimonious habitat suitability model [9]. Studies on cougars [62], jaguars (*Panthera onca*) [63] and leopards [64] have similarly found rugged topography to be important habitat for large predators. Our study area encompassed the highest peak (Letjume) in the western Soutpansberg Mountains [23] with significant on-site altitudinal variation [65,66]. As a consequence the region may constitute prime leopard habitat in terms of topography. Montane areas are difficult for humans to access, have lower human activity than less rugged terrain and thus offer isolated habitat with less direct competition for space and lower anthropogenic persecution than in lower lying areas [9]. Little or no cattle or game farming occurs in the highest elevations in the study area [55] further reducing the anthropogenic pressure and increasing the suitability of the habitat for leopards. 

Finally, land use within the Western Soutpansberg appears compatible with leopard conservation. Land use consists of private cattle and game farms, ecotourism properties, conservancies and communal farm land [55]. The camera trapping survey covered seven properties and an attitudinal survey of landowner perceptions towards leopards found that the majority of landowners (71%) who were engaged in consumptive and non-consumptive use of leopards (ecotourism and game farms) reported positive attitudes towards leopards [55]. Previous studies have also found landowners belonging to conservancies and eco-tourism operators to hold more positive attitudes to carnivores than those involved with livestock farming [67]. Although landowners engaged in cattle and small-stock farming have been found to be responsible for the bulk of leopard persecution in the area [55], as well as being one of the main causes of habitat fragmentation and persecution of leopards due to human wildlife conflict [9], such properties were at a low density in the upper reaches of the mountains [55]. As a consequence, land use in the study area may be more compatible with leopard conservation despite the potential influence of edge effects caused by the mosaic of land use types [7].

### Attractive sink or population source?

High density populations are often assumed to be population sources [68], but this conclusion ignores the possibility that areas with high population numbers may be acting as attractive sinks. Attractive sinks contain high numbers of dispersing sub-adults that enter an area with favourable habitat and unoccupied territories made vacant due to factors such as overharvesting [69]. Large numbers of dispersers can artificially inflate population numbers making the area appear to be a population source [70]. This short term inflation of population numbers can mask an overall metapopulation decline as dispersers are drawn into the sink and are then affected by the high mortality rates acting there. Large numbers of sub-adult individuals within a population are thus indicative of high population turnover and suggest a region is acting as an attractive sink.

Fourteen individual leopards were identified in this study of which ten were adults and four juveniles (Table 1). Due to the difficulty in aging leopards from camera trap photographs [41], sub-adults [2-3 years] and males less than 7 years were not classified in separate age groups. Although the demographic information presented in Table 1 could suggest a stable population, data from long-term (5 years) unpublished camera trapping records from the same area indicates only one of the adult males (AM2) has been continually present. This may imply a high turnover of sub-adult males and males under 7 years and so suggests that the area is functioning as a potential attractive sink. However, longer term data on population density and dynamics and the categorisation of individuals into narrower age classes is required in order to confirm this hypothesis. 

### Methodological considerations

The density estimate obtained via SECR (10.7 leopards per 100km^2^) is high in comparison to leopard densities calculated from other mountainous areas (Table 4). It is important to recognise however, that certain factors in survey design and analysis may bias density estimates. Recent research has shown that population estimates in SECR can be positively biased by the use of small camera trapping polygons [71-73]. Tobler & Powell [73] suggested that camera trapping polygons should be at least the size of the largest home range of a male in order to prevent bias from small survey size. During the course of our study, no data were available on the home ranges of adult males. A subsequent GPS satellite telemetry study provided home range data for one adult female (16.3km^2^ 95% kernel estimate) [55]. If no overlap occurred between females and an adult male home range overlapped with approximately four females [56], a male range might extend to 65km^2^. Our camera polygon of 31km^2^ was thus small compared to the recommendation of Tobler & Powell [73] and this may have positively biased the density estimate. Nevertheless, SECR models can produce unbiased density estimates even when the camera polygon size is about half the size of a home range of a male jaguar [73,74] and further work will be required to confirm the stability of our estimate.

A second consideration with our density estimate is the absence of sex covariates in the SECR analysis. Currently there is no facility in SPACECAP to use covariates, but sex and age can influence the movement and detection parameters, and therefore density [72,73]. Heterogeneous capture probabilities between males and females biases camera trapping data towards the sex with the greater encounter probability, in this case males [71], and this creates a negative bias in both traditional mark-recapture and spatial capture-recapture models leading to an underestimate of density [73]. One option to remove this bias is to estimate density separately for males and females, but this is only possible if sample sizes are adequate [71] which was not the case for our study (N=10 adult leopards). Nevertheless, since SECR models with sex covariates have shown that not accounting for sex could result in an underestimation of the true density estimate [72], future studies could improve the precision of density estimates through the inclusion of sex covariates.

Finally, research has shown that estimating the age of leopards based on photographs is difficult and can lead to misclassification of individuals to age groups [41], particularly for sub-adults (2-3 years of age). As a consequence, density estimates may be biased by the classification of some juveniles as adults, thus inflating the density estimate. Although based on our methods we are confident that all individuals were aged and sex correctly, incorrect age estimation remains an area of potential bias. If some juveniles were misclassified as adults, this could imply a population made up of a greater proportion of younger individuals, again suggesting that the survey site may be functioning as an attractive sink for immigrating individuals. Longer term data on population density and dynamics of known-age individuals are required to explore this further.

### Conservation and management implications

Where leopard populations are subject to illegal hunting, poaching and snaring, or legal harvesting through trophy hunting, increased hunting pressure will lead to a high risk of long-term decline. Wildlife authorities therefore need to take leopard population dynamics into account when assigning trophy hunting permits. Mortality from trophy hunting is additive to other forms of anthropogenic mortality. A recent call has been made to increase trophy hunting permits for leopards in the western Soutpansberg by local hunting factions who see the area as being a high density source for leopard trophies [55]. 

The results of this study provide evidence of a high density estimate of leopards in the afro-montane forest habitat of the western Soutpansberg Mountains (10.7 per 100 km^2^). This density estimate is the highest adult leopard population density recorded for a non-protected area in Africa (Table 4). Long term data are now needed on leopard densities over a larger area in the western Soutpansberg in order to confirm the conservation importance of this area. Analysis of these data must include the use of sex covariates, a survey area size that does not positively bias density results and the use of data on known-age individuals. Until these data are available calls to increase trophy hunting permits in the Soutpansberg should be resisted to ensure that the leopard population is not being over-harvested. An improvement in livestock husbandry practices in the study site is also required to mitigate human-wildlife conflict and illegal carnivore persecution and reduce the negative effects of anthropogenic persecution on leopard population dynamics [75]. 

## Conclusions

The western Soutpansberg Mountains in South Africa are home to the highest density of leopards recorded outside a state-protected area in sub-Saharan Africa. As leopards have experienced a 36.7% range loss across Africa, and only 20% of South Africa now holds suitable habitat for leopards, this mountain population may be of conservation importance for the wider leopard metapopulation [5,12]. Research has shown that unprotected, mostly privately owned land is extremely important for South African leopard conservation and in Limpopo Province 95% of suitable leopard habitat is still situated outside protected areas [12]. Leopard conservation efforts should therefore be focused on private land such as the Soutpansberg Mountains in order to preserve the status of this large carnivore.
